# Fighting Misconceptions to Improve Compliance with Influenza Vaccination among Health Care Workers: An Educational Project

**DOI:** 10.1371/journal.pone.0030670

**Published:** 2012-02-06

**Authors:** Carla R. Couto, Cláudio S. Pannuti, José P. Paz, Maria C. D. Fink, Alessandra A. Machado, Michela de Marchi, Clarisse M. Machado

**Affiliations:** Virology Laboratory (LIM 52 - HCFMUSP), Institute of Tropical Medicine, University of São Paulo, São Paulo, Brazil; College of Medicine, Hallym University, Republic of Korea

## Abstract

The compliance with influenza vaccination is poor among health care workers (HCWs) due to misconceptions about safety and effectiveness of influenza vaccine. We proposed an educational prospective study to demonstrate to HCWs that influenza vaccine is safe and that other respiratory viruses (RV) are the cause of respiratory symptoms in the months following influenza vaccination. 398 HCWs were surveyed for adverse events (AE) occurring within 48 h of vaccination. AE were reported by 30% of the HCWs. No severe AE was observed. A subset of 337 HCWs was followed up during four months, twice a week, for the detection of respiratory symptoms. RV was diagnosed by direct immunofluorescent assay (DFA) and real time PCR in symptomatic HCWs. Influenza A was detected in five episodes of respiratory symptoms (5.3%) and other RV in 26 (27.9%) episodes. The incidence density of influenza and other RV was 4.3 and 10.8 episodes per 100 HCW-month, respectively. The educational nature of the present study may persuade HCWs to develop a more positive attitude to influenza vaccination.

## Introduction

The compliance with influenza vaccination has been historically poor among health care workers (HCWs) varying from 2 to 36% around the world [1]–[4]. A recent review of relevant predictor studies of self-reported reasons for accepting or rejecting influenza vaccination showed that the two major reasons for rejecting are misconceptions or lack of knowledge about influenza infection; and a lack of convenient access to vaccine. On the other hand, HCWs compliant to seasonal vaccination are generally older, believe in vaccine efficacy, take the vaccine for self-protection, and have been previously vaccinated [5].

At Hospital das Clinicas, University of São Paulo School of Medical Sciences, a previous study showed a 34% compliance with influenza vaccination among HCWs. In the mentioned study, the main reasons for non-compliance were the perception of vaccine inefficacy and the fear of adverse reactions [4].

Respiratory symptoms occurring after vaccination are frequently misinterpreted as vaccine failure which reinforces the HCW's skepticism on vaccine efficacy. To overcome these false beliefs, we proposed a prospective study in a cohort of HCWs to demonstrate that influenza vaccine is safe and other respiratory viruses (but not influenza) are generally the cause of respiratory symptoms in the months following influenza vaccination.

## Materials and Methods

### 1. Setting

This study was conducted at Hospital das Clinicas, University of São Paulo School of Medical Sciences (HC-FMUSP) from May to October 2006. The Hospital das Clinicas is a 2,000-bed tertiary teaching hospital consisting of 5 buildings attached to the University of São Paulo. The main building has approximately 900 beds and contains most of the surgical and clinical wards and 12 intensive care units. Hospital das Clinicas has an estimated 15,000 HCWs, including permanent and casual staff, employees, students, and volunteers.

### 2. Influenza vaccination policies

Since 1999, annual influenza vaccination has been offered free of charge to all HCWs. Vaccination usually takes place at the hospital's Immunization Center during working hours, from Monday to Friday. In 2006, as a strategy to increase compliance with influenza vaccination, vaccine was offered at places of easy access during expanded hours, as suggested by 61% of the interviewed in previous survey [4]. In addition, an educational campaign was carried out emphasizing the safety and importance of influenza vaccination. Detailed information about the 2006 educational and vaccination campaign have been published elsewhere [6]. The HCW vaccination campaign was conducted from April 24 to May 8, 2006 and during this period 5,912 health professionals were vaccinated. The vaccine composition was A/New Caledonia/20/99 (H1N1) - like virus, A/California/7/2004 - (H3N2) - like virus and B/Malaysia/25/06/2004 - like virus.

### 3. Study design

During vaccination campaign, HCWs were invited to participate in the present study which had two steps: 1) evaluation of vaccine safety and, 2) cohort study to evaluate which respiratory viruses were more frequently detected in HCWs presenting respiratory symptoms in the four-month period following vaccination. Sample size was estimated taking into account an expected frequency of 10% of adverse events in adult population. Considering an acceptable frequency rate of up to 13%, we estimated to enroll at least 377 HCWs (EpiInfo version 6).

### 4. Study population

**Figure 1:**
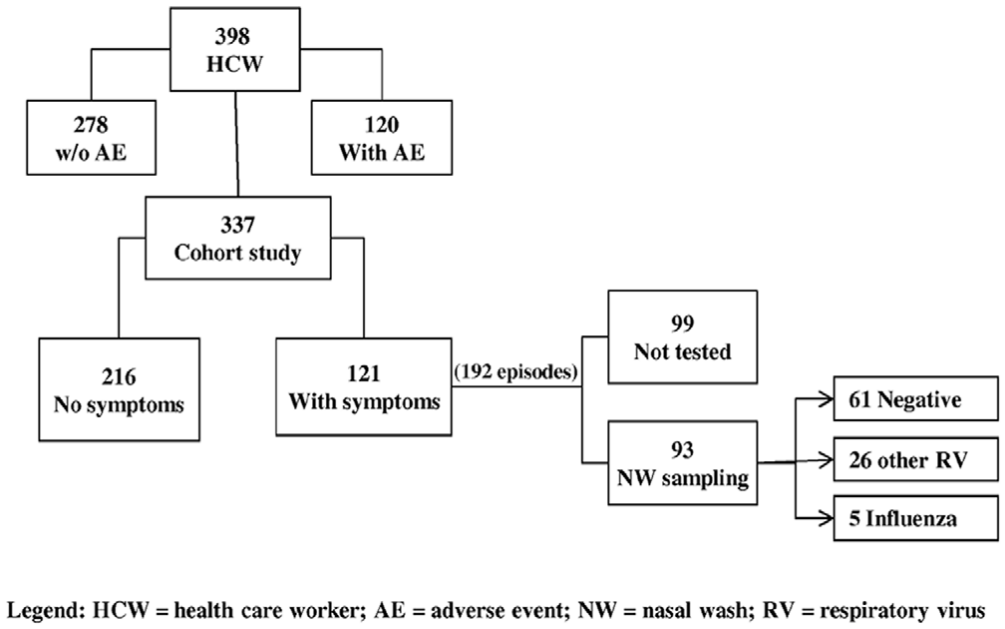


Three hundred and ninety eight vaccinated HCWs were surveyed for adverse events occurring within the first 48 h after influenza vaccination. A subset of 337 HCWs participated in the follow-up phase of the study. To assure that all hospital sectors were represented, the cohort was defined during the assessment of adverse events which was performed by a hospital epidemiologist nurse, through visits in all hospital floors and sectors. Afterward, these HCWs were actively surveyed twice a week, at work place, during four months, to check for the occurrence of respiratory symptoms and nasal wash sampling which was done in 93 of the participants. Figure 1 shows the algorithm of the study. A follow-up time of four months was proposed taking into account the period when serum antibodies elicited by influenza vaccine are expected to maintain protective levels.

### 5. Surveillance of adverse events and respiratory symptoms

The following adverse events were actively surveyed: fever, headache, malaise, myalgia, local pain, local edema and allergic reaction. Other adverse events spontaneously reported were also registered. During follow-up visits, participants were asked about the presence of the following symptoms: fever, coryza, blocked nose, sneeze, cough, watery eyes, headache, myalgia, sore throat, hoarseness, sibilance, and dyspnea. Allergy was ruled out in those with sneezing as the only symptom. Influenza like illness (ILI) was defined by the presence of fever and cough and/or sore throat according to the CDC definition [7]. In the presence of any of the above mentioned symptoms, a nasal wash sample was taken according to Englund et al, kept at 4°C to 8°C and processed at the virology laboratory within four hours from sampling [8]. Nasal washes were taken from HCWs who consent with sampling and whose duration of symptoms did not exceed three days.

### 6. Respiratory virus diagnosis

Respiratory syncytial virus (RSV), influenza (INF) A and B, adenovirus (ADV) and parainfluenza virus (PIV) were diagnosed by direct immunofluorescent assay (DFA) according to the manufacturer's instructions (Imagen® DAKO, Cambridgeshire, UK). Aliquots of NW samples were stored at −80°C for later PCR and real time PCR processing. PCR was used to detect coronavirus and picornavirus. RT-PCR products for picornavirus were subsequently sequenced to differentiate rhinovirus from enteroviruses. Real time PCR (Taqman assay) was used to diagnose human metapneumovirus (hMPV). To increase the sensitivity of influenza diagnosis, a real time PCR (Taqman assay), was added to the diagnostic tools. Similarly, a nested adenovirus PCR was used along with DFA due to the low sensitivity of the latter in diagnosing ADV. The PCR protocols used in the present study have been published elsewhere [9]–[12]. HCWs were informed about the results of the DFA up to 48 h after sampling.

### 7. Ethics Statement and informed consent

The study was approved on 04/12/2006 by local Ethics Committee with the following statement: “*A Comissão de Ética para análise de projetos de Pesquisa - CAPPEsq da Diretoria Clínica do Hospital das Clínicas e da Faculdade de Medicina da Universidade de São Paulo, em sessão de 12/04/2006, aprovou o Protocolo de Pesquisa no. 241/06, intitulado Viroses respiratórias após vacinação contra influenza em profissionais de saúde, apresentado pelo Departamento de Moléstias Infecciosas e Parasitárias, inclusive o Termo de Consentimento Livre e Esclarecido. CAPPesq, 12/04/2006*”. Written informed consent was obtained from all participants.

### 8. Statistical analysis

SPSS 15.0 (SPSS Inc., Chicago, IL) was used with the *χ* ^2^ test or Fisher's exact test for discrete variables, and Student's t test or the Mann-Whitney U test for continuous variables. Tests of significance were two sided, and p<0.05 was considered to be statistically significant. Incidence density (ID) of respiratory symptoms, influenza virus infections and other respiratory virus infections were calculated by the formula described below. ID results were expressed *per* 100 HCW-month [13].
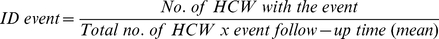



## Results

The interviews for adverse events occurring within 48 h of vaccination were made from one to 10 days after vaccination. The majority of the participants (81.4%) were surveyed within the first week of vaccination. All hospital sectors were represented (Table 1). One hundred and twenty of the 398 HCWs (30.2%) reported at least one adverse event (AE). Table 1 shows the occurrence of adverse events according to the demographic characteristics of the vaccinees. Sector of work was the only variable associated with the presence of adverse events (*p* = 0.017). The sectors with highest frequency of adverse events were the Virology Laboratory (87.5% of the subjects), Burn unit (54.5%), Nephrology (52.4%) and Pneumology (45.5%). In the remaining sectors, AE were reported by less than 40% of the subjects. Those surveyed in the first five days after vaccination were more likely to report such events [95 (79.2%) *versus* 25 (20.8%); p<0.0001]. Local AE were reported by 18.3% of the participants, systemic AE by 71.6%, and 10% of the participants reported both local and systemic AEs. Headache, myalgia and malaise were more frequently reported (50%, 45.8% and 45%, respectively). Local pain and local edema was reported by 17.5% and 5% of the HCWs. No severe adverse event was observed.

**Table 1:** Demographic characteristics of HCWs surveyed for adverse events (AE).

Variable	Category	Distribution (%)	Number (%)		*p* value
			Without AE	With AE	
Age* (years)	18–35	195 (49.5)	138 (70.8)	57 (29.2)	0.84
	36–80	199 (50.5)	139 (69.8)	60 (30.2)	
Gender	Male	42 (10.6)	30 (71.4)	12 (28.6)	0.81
	Female	356 (89.4)	248 (69.7)	108 (30.3)	
Sector	Emergence room	56 (14.0)	40 (71.4)	16 (28.6)	0.017
	General surgery	42 (10.6)	33 (78.6)	09 (21.4)	
	Internal medicine	34 (8.5)	30 (88.2)	04 (11.8)	
	Pneumology	22 (5.5)	12 (54.5)	10 (45.5)	
	Nephrology	21 (5.3)	10 (47.60	11 (52.4)	
	Neonate unit	19 (4.8)	13 (68.4)	06 (31.6)	
	Neurology	18 (4.5)	12 (66.7)	06 (33.3)	
	Hematology/HSCT	17 (4.3)	13 (76.5)	04 (23.5)	
	Maintenance	17 (4.3)	11 (64.7)	06 (35.3)	
	Liver transplantation	15 (3.8)	09 (60)	06 (40)	
	Obstetrics/Gynecology	14 (3.5)	12 (85.7)	02 (14.3)	
	Infectious diseases	13 (3.3)	11 (84.6)	02 (15.4)	
	Operating room	12 (3.0)	09 (75)	03 (25)	
	Burn unit	11 (2.8)	05 (45.5)	06 (54.5)	
	Renal transplantation	10 (2.5)	06 (60)	04 (40)	
	Geriatrics	09 (2.3)	06 (66.7)	03 (33.3)	
	Virology laboratory	08 (2.0)	01 (12.5)	07 (87.5)	
	Ophthalmology	05 (1.3)	04 (80)	01 (20)	
	Dermatology	05 (1.3)	05 (100)	0 (0)	
	Urology	05 (1.3)	04 (80)	01 (20)	
	Rheumatology	05 (1.3)	03 (60)	02 (40)	
	Vascular surgery	05 (1.3)	04 (80)	01 (20)	
	Other	35 (8.8)	25 (71.4)	10 (28.6)	

(*) Information not available in 4 subjects. HSCT = hematopoietic stem cell transplantation.

During the 4-month period following influenza vaccination, respiratory symptoms were evaluated in 337 HCWs. A total of 4,182 follow-up visits were performed (median 12 *per* HCW, ranging from one to 25 visits).

One hundred and twenty-one HCWs (36%) developed 192 episodes of respiratory symptoms. Coryza, cough, sore throat and myalgia were reported by 36.3%, 25%, 17.4% and 13.7% of the participants, respectively. Seventy-one of them (58.7%) presented more than one episode suggestive of upper respiratory infection (URI). ILI was observed in 17 of the 192 episodes (8.8%). Mean time to the occurrence of respiratory symptoms was 2.9 (0.7 to 5.2) months. The incidence density of respiratory symptoms was 12.4 episodes *per* 100 HCW-month.

Nasal washes were taken in 93 of the 192 episodes of URI. In 61 episodes (66.3%) no respiratory virus was found, even though 82% had coryza, 53% had cough and 49% had sore throat. The frequency of ILI was similar among HCWs who agreed with sampling and those who did not agree (58.8% *versus* 41.2%, *p* = 0.37) (Table 2).

**Table 2:** Influenza-like illnesses in HCWs according to acceptance of nasal wash sampling.

Influenza-like illness	Agreed with sampling (%)		Total
	No	Yes	
No	92 (47.9)	83 (43.2)	175 (91.1)
Yes	07 (3.6)	10 (5.2)	17 (8.9)
Total	99 (51.6)	93 (48.4)	192 (100)

(*p* = 0.37; Pearson χ^2^ test).

Influenza A virus was detected in 5 of 93 episodes (5.4%), considering both techniques. DFA diagnosed two cases and real time PCR detected three additional ones. Respiratory symptoms in HCWs with influenza are described on table 3. Influenza cases occurred at a median of 40 days after vaccination, ranging from 34 to 56 days and were considered vaccine failures. Mean time to influenza diagnosis was 1.24 months. Incidence density of influenza was 4.3 cases *per* 100 HCW-month.

**Table 3:** Time to influenza diagnosis according to vaccination date of HCWs and respective workplaces.

Case	Influenza symptoms	Vaccine date	Influenza diagnosis	Δt	Sector
1	Cough, sneezing, coryza	04/27/06	05/31/06	34	Internal Medicine
2	Fever, sore throat	04/26/06	06/01/06	36	Urology
3	Nasal congestion, cough, myalgia, sibilance	04/27/06	06/06/06	40	Internal Medicine
4	Coryza, headache	04/26/06	06/06/06	41	Trauma ICU
5	Fever, coryza, headache, nasal congestion, myalgia	04/25/06	06/20/06	56	Nephrology

Other respiratory viruses were diagnosed in 26 of the 93 episodes (28.3%) of URI. Rhinovirus and coronavirus were the RV more frequently detected (Table 4). Mean time to the occurrence of other RV infections was 2.72 months. Incidence density of other RV was 10.3 cases *per* 100 HCW-month. Other RV infections occurred significantly later than influenza cases during follow-up (*p* = 0.04).

**Table 4:** Other respiratory viruses diagnosed by DFA and/or PCR/real time PCR.

Respiratory Virus	No. tested positive	%
Respiratory syncytial virus	1	3.2%
Adenovirus	2	6.5%
Coronavirus	7	22.5%
Influenza A	5	16.1%
Metapneumovirus	3	9.6%
Rhinovirus	9	29.0%
Adenovirus+Coronavirus	1	3.2%
Metapneumovirus+Rhinovirus	1	3.2%
Rhinovirus+Coronavirus	1	3.2%
Respiratory syncytial virus+Adenovirus	1	3.2%
TOTAL	31	100.0%

## Discussion

Misconceptions about influenza vaccine, be it about its safety or its effectiveness, have been identified in all studies included in a recent review of attitudes and predictors of influenza vaccination among HCWs, highlighting the importance of education efforts [5].

Initial symptoms of RV infections are often unspecific such as fever, malaise, or myalgia. As influenza vaccine is offered when other RV are circulating (e.g., RSV), vaccinated HCWs developing symptoms within 48 h of vaccination misinterpret those signs as vaccine adverse events. In addition, the occurrence of respiratory symptoms in the months following vaccination is mistaken as vaccine failure. Other respiratory infections as the cause of such symptoms are hardly ever considered.

To diminish the arguments of fear of adverse events or perception of vaccine inefficacy, this prospective study was conducted to demonstrate to a subset of HCWs from our hospital, that severe adverse events following influenza vaccination are rare and the episodes of respiratory symptoms occurring in the first four months after vaccination are generally caused by other respiratory viruses and not by influenza virus.

As expected, no severe adverse event was observed in the present study, and the events more frequently reported, such as headache, myalgia and malaise could be related to influenza vaccine itself as well as to other causes, given their unspecificity.

In adults, the adverse event more frequently reported after intramuscular administration of inactivate vaccines is local pain, affecting 10% to 64% of the vaccinated [14]–[16]. In the present study, 17.5% of the participants reported local pain.

Systemic reactions like fever, malaise and myalgia can also occur after inactivate vaccines. In the present study, the frequency of systemic AEs (over 70%) was higher than reported in previous studies. Recent publications have shown rates of systemic adverse events ranging from 30% to 59% in HWCs [17], [18]. Ideally, the subjects should have been surveyed within the first four days of vaccination. As we preferred to apply the questionnaire personally, rather than by mail or phone calls, only 49.7% of the participants were interrogated up to the fourth day, due to the great number of interviews. Thus, the high rate of systemic adverse events observed in the present series may be either an overestimation by the subjects or a consequence of the survey method applied. A recent study evaluating vaccine coverage in Korea has demonstrated that interview surveys provide more reliable information than telephone surveys, showing lower missing rates and 100% of agreement with the immunization registry record [18].

Anaphylaxis and neurological reactions are rare [15], [19]. The frequency of adverse events observed in the present study may be overestimated taking into account the subjectiveness of self-reported unspecific symptoms.

As HCWs are aware of vaccine adverse events and fear its consequences, it is comprehensible that these events will be more frequently reported by them than by general population. Another study conducted in the same hospital demonstrated that HCWs reported significantly more adverse events (52.9%) than the elderly (25.3%) [20]. The higher frequency of adverse events reported by HCWs surveyed in the first five days of vaccination, as compared with those surveyed after the fifth day, may suggest that people may be more predisposed to remember any symptom possibly associated with the vaccine if inquired within the first days of vaccination. On the other hand, we believe that if the adverse events were severe or important, they would not be missed if inquired after 6 to 10 days.

Interestingly, we observed that some sectors showed significantly higher rates of AE than others, supporting the subjectivity of the information. Also, this data may suggest a mouth to mouth effect among sector coworkers influencing the self-report of AE. Among HCWs, the belief that coworkers take influenza vaccine influences the vaccine uptake. Thus, it is possible that the same occurs concerning to adverse events.

Continued education of health professionals is essential to highlight not only the epidemiological importance of the vaccine, but also its safety and the low risk of severe adverse events.

Our study also demonstrated that the respiratory symptoms occurring in the months following influenza vaccination were more frequently caused by other respiratory viruses and generally do not mean vaccine failures.

One limitation of our study is that in only 93 of the 192 episodes of respiratory symptoms (48.4%) the subjects agreed with NW sampling. NW sampling is a simple but uncomfortable procedure and this fact may explain why some HCWs preferred not to get tested during working hours. One could argue that influenza cases could be missed among those not tested. However, we believe that this loss has not affected our results as the frequency of ILI was similar between those who agreed with sampling and those who did not (Table 2, *p* = 0.37).

The incidence density of other respiratory viruses was 2.4 times greater than incidence density of influenza. Probably, this difference would be even greater if real time PCR was also performed to increase the sensitivity of the diagnosis of other respiratory viruses as well. In addition, more cases of other RV infections would be diagnosed if a larger number of professionals were tested, increasing the difference between the incidence density of influenza and other RV.

Influenza infection is characterized by the abrupt occurrence of fever, headache, myalgia, and dry cough. During influenza season, the presence of these symptoms is highly predictive of influenza infection and summarizes the case definition of *influenza-like illness* (ILI), which has been used worldwide for influenza surveillance purposes. However, the sensitivity and positive predictive value of such definition can vary greatly depending on the co-circulation of other respiratory viruses in the community [21]. Indeed, Bellei et al. have recently reported that 70% of ILI cases in the city of São Paulo were caused by other agents, mainly rhinovirus, which peaks along with influenza [22]. Similar results have been previously published by other authors [21].

In our series, influenza cases in vaccinated HCWs were mild and occurred significantly earlier following vaccination in comparison to other respiratory viruses. This finding may be explained by the marked seasonality of influenza in São Paulo city as reported previously [23], [24], peaking in early winter and coinciding with the initial period of the study.

The effectiveness of influenza vaccines is related predominantly to the age and immune competence of the vaccinee and the degree of similarity between the viruses in the vaccine and those in circulation. Vaccine effectiveness in preventing laboratory-confirmed influenza illness when the vaccine strains are well matched to circulating strains is 70–90% in randomized, placebo-controlled trials conducted among children and young healthy adults, but is lower among elderly or immunocompromised persons [25]. In adults ≥65 years old, the efficacy of influenza inactivate vaccine varies from 30% to 40% [26].

Trials that measure laboratory-confirmed influenza virus infections as the outcome are the most persuasive evidence of vaccine efficacy [25]. In the present study, only five of the 337 vaccinated HCWs (1.5%) acquired influenza.

In accordance with the educational nature of our study, we considered all cases of influenza as vaccine failures, since vaccinated health personnel look forward to be protected against influenza. Molecular characterization of influenza cases was not performed to check for possible mismatches between circulating viruses and vaccine strains, which could possibly justify those failures.

Our study demonstrated that the fear of severe adverse events seems unjustified as well as the perception of vaccine inefficacy. URI following influenza vaccination were generally caused by other respiratory viruses and not by influenza.

In times of pandemic influenza A H1N1 and widespread vaccination, healthcare and emergency medical services personnel are among the priority groups recommended to receive the H1N1 influenza vaccine. It is time to overcome definitively the misconceptions about the vaccine as well as the fear of adverse events. So far, the vast majority (93%) of adverse events reported to VAERS after receiving the trivalent 2010–2011 influenza vaccine, were classified as “non serious”, e.g., soreness at the vaccine injection site [27].

We believe that the educational nature of the present study may persuade HCWs to develop a more positive attitude to influenza vaccination.
